# Regulation of TFPIα expression by miR-27a/b-3p in human endothelial cells under normal conditions and in response to androgens

**DOI:** 10.1038/srep43500

**Published:** 2017-02-27

**Authors:** Ana B. Arroyo, Salam Salloum-Asfar, Carlos Pérez-Sánchez, Raúl Teruel-Montoya, Silvia Navarro, Nuria García-Barberá, Ginés Luengo-Gil, Vanessa Roldán, John-Bjarne Hansen, Chary López-Pedrera, Vicente Vicente, Rocío González-Conejero, Constantino Martínez

**Affiliations:** 1 Centro Regional de Hemodonación, University of Murcia, IMIB-Arrixaca, Murcia, Spain; 2 Maimonides Institute for Research in Biomedicine of Cordoba (IMIBIC). Reina Sofia University Hospital, University of Cordoba, Cordoba, Spain; 3 Medical Research Institute, Hospital Universitario y Politécnico La Fe, Valencia, Spain; 4 K. G. Jebsen - Thrombosis Research and Expertise Center (TREC), Department of Clinical Medicine, UiT - The Arctic University of Norway, Tromsø, Norway; Division of Internal Medicine, University Hospital of North Norway, Tromsø, Norway

## Abstract

The increased risk of cardiovascular events in older men is multifactorial, but the significant reduction of testosterone levels has been involved. As this hormone regulates the expression of TFPI by unknown mechanisms, we aimed to evaluate the role of miRNAs in the regulation of TFPIα expression under normal conditions and in response to androgens. *In silico* studies allowed the selection of 4 miRNAs as potential TFPIα regulators. Only miR-27a/b-3p significantly reduced TFPIα expression in two endothelial cell lines. Luciferase assays demonstrated a direct interaction between miR-27a/b-3p and *TFPI* 3′UTR. *Ex vivo* analysis of *TFPI* and miRNA levels in 74 HUVEC samples from healthy subjects, showed a significant and inverse correlation between *TFPI* and miR-27a-3p. Moreover, anticoagulant activity of TFPIα from cells supernatants decreased ~30% with miR-27a/b-3p and increased ~50% with anti-miR-27a/b-3p. Interestingly, treatment of EA.hy926 with a physiological dose of dihydrotestosterone (30 nM) significantly increased (~40%) TFPIα expression with a parallel decreased (~50%) of miR-27a/b-3p expression. In concordance, increased levels of miR-27a/b-3p normalized the up-regulation induced by testosterone. Our results suggest that testosterone is a hinge in miR-27/TFPIα regulation axis. Future studies are needed to investigate whether testosterone variations are involved in a miR-27/TFPIα dysregulation that could increase the cardiovascular risk.

Tissue factor pathway inhibitor (TFPI), the natural and primary inhibitor of tissue factor (TF), is a prime candidate to control thrombosis through the regulation of TF^1^. Few studies have investigated the mechanisms implicated in TFPI regulation which may be of paramount importance to better define its biology and its role in thrombosis. The variability of TFPI plasma levels due to genetic factors account for almost 50%^2^,^3^,^4^,^5^, but little is known on other factors, in particular hormones.

During the last years, different groups including ours, have evaluated the role of miRNAs in the regulation of haemostasis^6^, as this regulation may have an impact on the thrombotic aetiology^7^. MicroRNAs (miRNAs), are small non-coding RNAs that regulate protein expression that have been involved in the regulation of many complex biological mechanisms^8^ and in many pathological conditions^9^,^10^. Thus, three evidences encourage a deep characterization of the testosterone-miRNA-TFPI triad as a new regulatory axis in endothelial function: (i) experimental studies have shown that testosterone up-regulates TFPI expression in endothelial cells^11^,^12^; (ii) low levels of testosterone are associated with low plasma levels of TFPI and an increase of cardiovascular risk^13^,^14^,^15^, and (iii) testosterone induces changes in miRNA expression pattern in prostate cancer cells^16^,^17^ or muscle^18^,^19^.

Therefore, the purpose of this study was to determine whether TFPIα is regulated by miRNAs through a testosterone-dependent mechanism.

## Results and Discussion

### miR-27a/b-3p directly bind and regulate the expression of TFPIα

We first sought to identify miRNAs as novel and specific regulatory elements in *TFPI* 3′UTR. Computational results from the different algorithms showed up 4 miRNA candidates: miR-27a/b-3p, miR-19b and miR-24 (Table 1).

**Figure 1:**
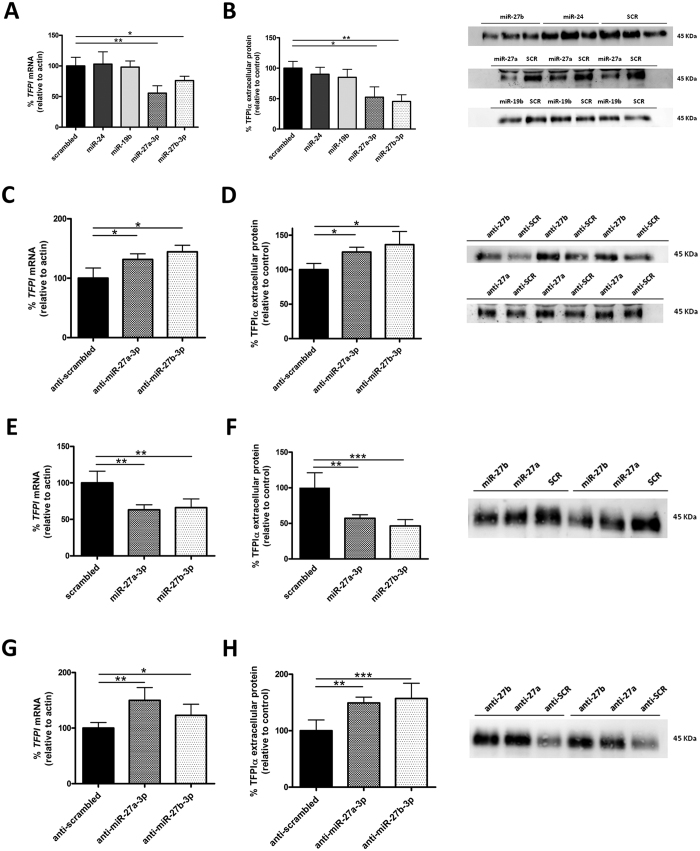
miR-27a/b-3p directly inhibit the expression of TFPIα. Endothelial cells were transfected with different miRNA precursors (100 nM) selected by *in silico* prediction or a scrambled control and mRNA levels were measured after 48 h by qRT-PCR in EA.hy926 (**A**) and in HUVECs (**E**). Protein levels in cell culture supernatants were measured by densitometry after western blotting with specific anti-TFPI antibody in EA.hy926 (**B**) and in HUVECs (**F**). A representative blot is shown. The TFPI mRNA in EA.hy926 (**C**) and in HUVECs (**G**) and the respective protein levels (**D**,**F**) of cells transfected with miR-27a/b-3p inhibitors, and determined by the same methods and in the same conditions, is also shown. The 2^−ΔCt^ method was followed to calculate the relative abundance of mRNA compared with endogenous control expression of β-actin (*ACTB*) (Ct = Threshold Cycle; ΔCt = Ct sample gene - Ct endogenous control). For miR-27b and miR-24 the same scrambled control was used. Representative blots are shown. All results are represented as mean ± SD from at least three experiments performed in triplicate (*p < 0.05; **p < 0.01; ***p < 0.001).

**Figure 2:**
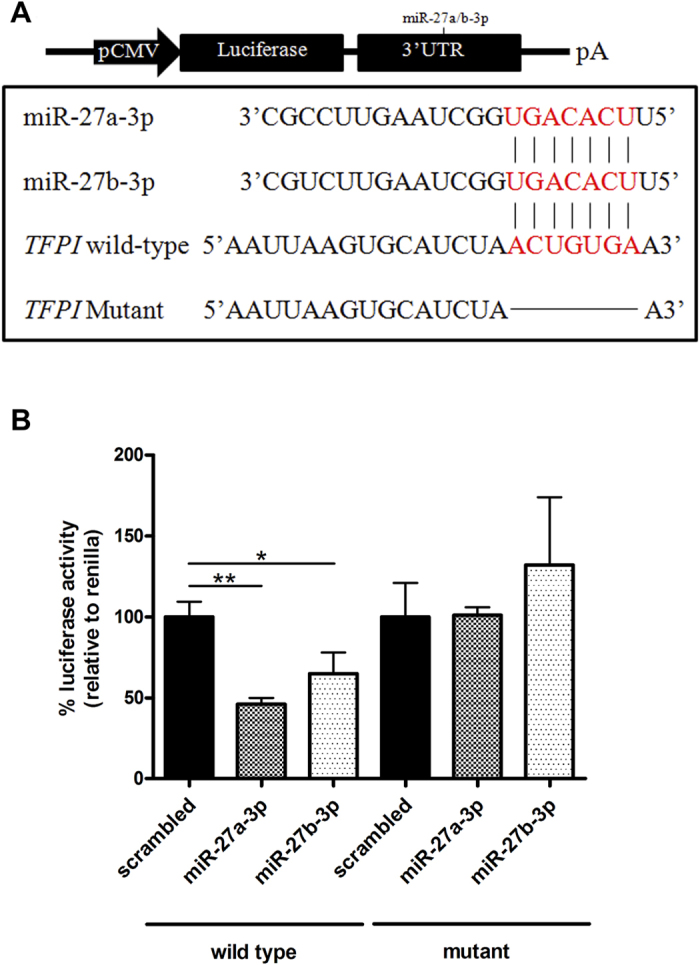
miR-27a/b-3p directly target *TFPI* mRNA. (**A**) Schematic diagram of the luciferase reporter. Plasmids including *TFPI* WT 3′UTR or *TFPI* mutant 3′UTR in which the seven nucleotides forming the seed region of miR-27a/b-3p were deleted. (**B**) HTC-DK cells were transfected with either *TFPI* WT 3′UTR or *TFPI* mutant 3′UTR along with 100 nM miR-27a/b-3p precursor. A scrambled precursor was used as control. Luciferase activities were normalized to renilla activities. The 2^−ΔCt^ method was followed to calculate the relative abundance of mRNA compared with endogenous control expression of β-actin (*ACTB*) (Ct = Threshold Cycle; ΔCt = Ct sample gene - Ct endogenous control). All results are represented as mean ± SD from at least three experiments performed in triplicate (*p < 0.05; **p < 0.01).

We validated *in silico* results by transfecting EA.hy926 endothelial cells (ECs) with all the miRNA candidates. Only miR-27a/b-3p mimics produced a decrease in both *TFPI* mRNA and extracellular TFPIα levels (Fig. 1A and B), as well as anti-miR-27a/b-3p produced an increase in *TFPI* mRNA and extracellular TFPIα levels (Fig. 1C and D). These results were confirmed in another EC type (Fig. 1E–H). To reveal whether the effect of miR-27a/b-3p on TFPIα was direct or indirect, we cloned a fragment of *TFPI* 3′UTR (NM_006287) into a luciferase vector. As a negative control, we deleted the seed region for miR-27a/b-3p. MiR-27a/b-3p significantly reduced the luciferase activity, whereas no effect was observed with the mutated luciferase vector (Fig. 2). Thus, we here described for the first time that TFPIα is directly regulated by two miRNAs, miR-27a/b-3p in ECs and this post-transcriptional regulation of TFPIα might have consequences on the endothelial functions. Emerging evidences have already pointed toward miRNAs as relevant regulators of haemostatic proteins. For example, TF is regulated by miRNAs from the miR-17~92 cluster in monocytes^7^ and by miR-223 in ECs^20^. On the other hand, protein S (PS), a cofactor for full length TFPIα^21^, is regulated by miR-494^22^. Thus, we can speculate that the regulation of TFPIα, TF, and PS may be exerted by a miRNA hub that modulate the TF/TFPI axis, as it has been described for other signaling pathways^20^,^23^. Indeed, *in silico* analyses showed that miR-27a/b-3p could also target TF and PS, although these interactions have to be further demonstrated *in vitro*.

### miR-27a-3p inversely correlated with TFPI expression in HUVECs

**Figure 3:**
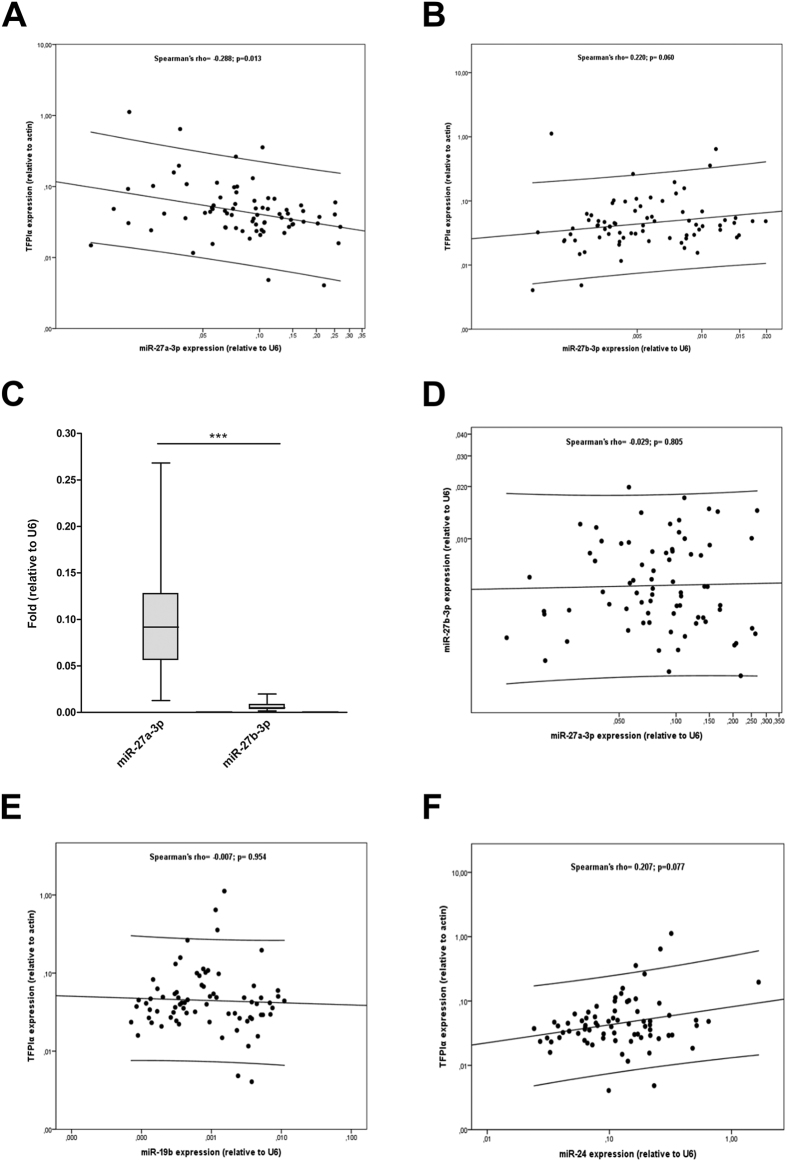
*Ex vivo* expression of *TFPI* mRNA and mature miRNAs. Levels of miR-27a-3p (**A**), miR-27b-3p (**B**), miR-19b (**E**) and miR-24 (**F**) were measured by qRT-PCR and correlated with *TFPI* mRNA expression in 74 HUVEC samples obtained from umbilical cords. (**C**) Mean levels of miR-27a/b-3p in HUVECs. (**D**) correlation between miR-27a-3p and miR-27b-3p. The 2^−ΔCt^ method was followed to calculate the relative abundance of miRNA compared with endogenous control expression of U6 (Ct = Threshold Cycle; ΔCt = Ct sample gene - Ct endogenous control) (**p < 0.01).

With the aim to test whether the TFPI:miR-27a/b-3p interaction has physiological consequences, we quantified these transcripts in 74 HUVEC samples (from healthy human donors). By using a correlation model, we found that *TFPI* mRNA and miR-27a-3p levels were inversely and significantly associated (Fig. 3A). However, we found a lack of agreement between the *in vitro* and *ex vivo* results for miR-27b-3p (Fig. 3B) that may be justified because its expression was 18-fold lower than miR-27a-3p in HUVECs (Fig. 3C), which is in accordance with published results^24^. Indeed, miR-27a and miR-27b were not correlated (Fig. 3D). Therefore it is expected that, in physiological conditions, the lower concentration of miR-27b-3p in HUVECs may explain its lack of association with TFPI levels and potentially a minor role in regulating TFPIα in ECs. As a control, we evaluated the correlations of miR-19b and miR-24 with *TFPI*. As expected, no significant correlations were observed (Fig. 3E and F), which strengthen the results obtained for miR-27a-3p. Accordingly, the quantification of miR-27a/b-3p might be of interest in pathological conditions where miRNA deficiency or over-expression may have an important impact^25^.

### miR-27a/b-3p levels modulate the anticoagulant effect of TFPIα

**Figure 4:**
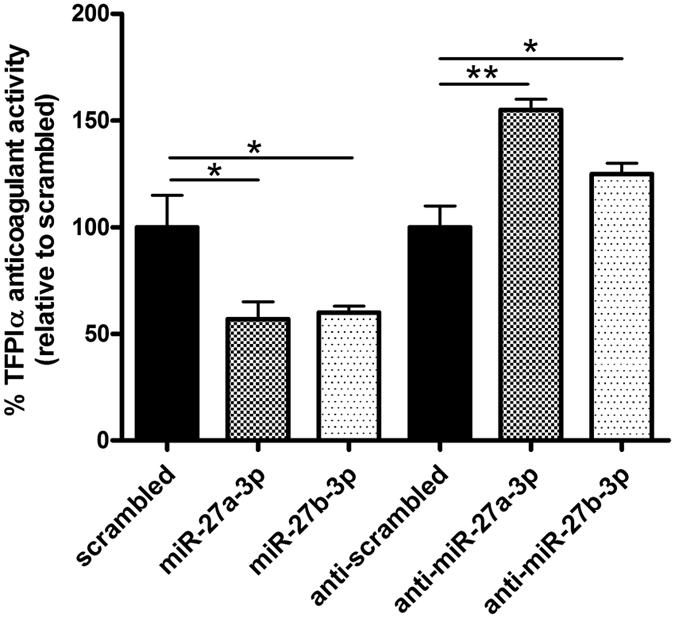
miR-27a/b-3p regulate the anticoagulant activity of TFPIα in HUVECs. TFPIα anticoagulant activity was measured in the supernatant from HUVECs transfected with precursors or inhibitors of miR-27a/b-3p. All results are represented as mean ± SD from at least three experiments performed in triplicate (*p < 0.05; **p < 0.01).

We next investigated the functional relevance of TFPIα regulation by miR-27a/b-3p. TFPIα anticoagulant activity was measured in the supernatant of HUVECs transfected with both miRNAs. Transfection with miR-27a/b-3p significantly decreased TFPIα anticoagulant activity, while anti-miR-27a/b-3p significantly increased TFPIα anticoagulant activity (Fig. 4). These latter observations were further confirmed in EA.hy926 cells transfected with miR-27a-3p (Figure S1). This finding suggests that miR-27a/b-3p levels variations may have an impact on TFPIα functionality. Hypoxic conditions have been described previously to up-regulate miR-27a/b-3p expression^26^ while infective and inflammatory processes down-regulate their expression^27^,^28^. Given the extensive cross-talk between the coagulation and inflammatory systems, TFPI included^29^, additional studies are certainly needed to reveal the role of miR-27a/b-3p in TFPIα regulation and the impact on their activity under pathological conditions.

### TFPIα regulation by testosterone is dependent of miRNA expresssion

**Figure 5:**
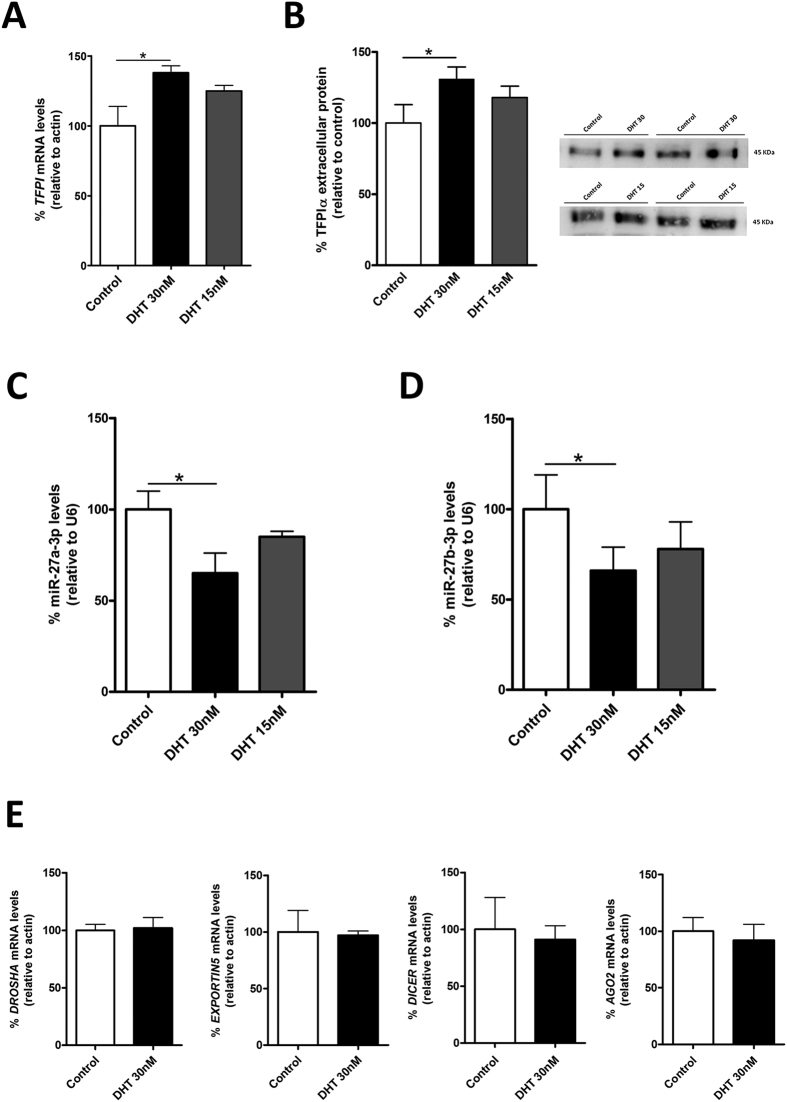
DHT regulates miR-27a/b-3p and TFPI expression. EA.hy926 cells were activated with physiological (30 nM) or low (15 nM) doses of DHT and *TFPI* mRNA (**A**), secreted TFPIα expression (**B**) and miR-27a/b-3p levels (**C**,**D**) were measured. (**E**) Levels of mRNA transcripts from enzymes implicated in the miRNA biogenesis: Drosha, Argonaute 2 (AGO2), Exportin 5 and Dicer were measured by qRT-PCR. The 2^−ΔCt^ method was followed to calculate the relative abundance of miRNA or mRNA compared with endogenous control expression of U6 or *ACTB* (Ct = Threshold Cycle; ΔCt = Ct sample gene - Ct endogenous control). All results are represented as mean ± SD from at least three experiments performed in triplicate (*p < 0.05).

It is described that testosterone increases *TFPI* mRNA and protein levels^11^,^12^. Nevertheless, the specific mechanisms of this regulation are largely unknown. In addition, testosterone may also regulate miRNA expression^16^,^19^. Thus, we wondered whether miR-27a/b-3p could be implicated in TFPI regulation by testosterone. We treated EA.hy926 cells with a physiological dose of the main testosterone metabolite, dihydrotestosterone (DHT, 30 nM) and we observed a significant increase of both *TFPI* mRNA (Fig. 5A) and protein after 24 h (Fig. 5B). Indeed, such doses of DHT decreased miR-27a/b-3p (Fig. 5C and D). These results were also shown in HUVECs (Figure S2). Key enzymes implicated in the miRNA biogenesis: Drosha, Ago2, Exportin 5, and Dicer were not significantly affected by DHT treatment (Fig. 5E). Therefore, regulation of miR27a/b-3p by testosterone seems to be specific and non-dependent of miRNA biogenesis machinery. Although it has been shown that testosterone increases the rate of pri-miR-27a maturation through the binding of the androgen receptor (AR) to the promoter of the gene in LNCaP prostate cancer cells^30^, our results showed that DHT provokes a decrease of miR-27a/b-3p in ECs. These results could be partially explained through differences of regulation pathways between both cell types that can certainly have an impact on the miR-27a/b-3p-testosterone regulation^31^. Future studies are needed to investigate whether testosterone variations are involved in a miR-27/TFPIα dysregulation that could increase the cardiovascular risk inherent to aging. However, an important limitation of forthcoming studies will be the accessibility to the adequate samples, *e.g*. ECs, that can be circumvented by the use of an appropriate animal model.

**Figure 6:**
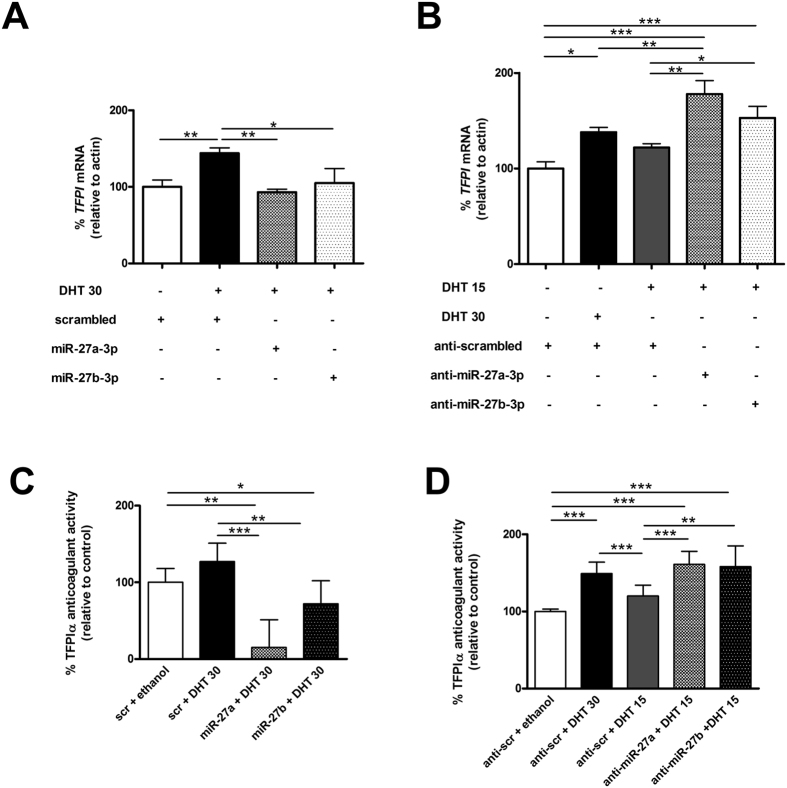
miR-27a/b-3p regulate DHT-dependent TFPI expression. EA.hy926 cells transfected with (**A**) miR-27a/b-3p precursors or (**B**) miR-27a/b-3p inhibitors were activated with 15 nM or 30 nM DHT. After 48 h levels of TFPI mRNA were measured by qRT-PCR as well as TFPI anticoagulant activity (**C**). The 2^−ΔCt^ method was followed to calculate the relative abundance *TFPI* mRNA compared with endogenous control expression of *ACTB* (Ct = Threshold Cycle; ΔCt = Ct sample gene - Ct endogenous control). All results are represented as mean ± SD from at least three experiments performed in triplicate (*p < 0.05; **p < 0.01; ***p < 0.001).

**Figure 7:**
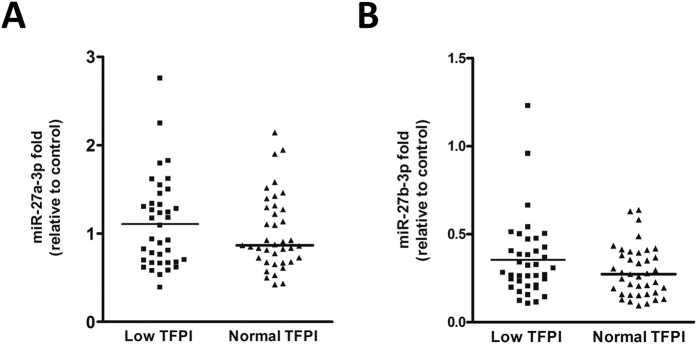
Levels of miR-27a/b-3p in human plasma. (**A**) miR-27a and (**B**) miR-27b measured in plasma samples of 70 year-old patients with low TFPI (n = 37; 10.9 ± 2.3 ng/L) expression and normal TFPI (n = 41; 12.3 ± 3 ng/L) expression^13^. The 2^−ΔCt^ method was followed to calculate the relative abundance of miR-27a/b compared with control (geometric mean of miR-103-3p and miR-191) (Ct = Threshold Cycle; ΔCt = Ct sample gene - Ct endogenous control). Lines indicate population median.

Finally, combination of androgen treatment with miR-27a/b-3p transfection (Fig. 6A and C) but not with miR-19b or miR-24 (Figure S3) in EA.hy926 cells abolished TFPI up-regulation produced by 30 nM DHT as well as the anticoagulant activity, showing a relationship between testosterone, TFPI, and miR-27a/b-3p. In fact, when ECs transfected with anti-miR-27a/b-3p where treated with 15 nM DHT, we observed a significant increase of TFPI mRNA (Fig. 6B) and of the anticoagulant activity (Fig. 6C) further demonstrating the potential involvement of miR-27a/b-3p in the regulation of *TFPI* levels in ECs. The effect of miR-27a/b-3p mimics were also shown in HUVECs (Figure S4). Thus, the assessment of therapeutic alternatives with miRNAs may be of interest. This model suggests that in ECs high levels of miR-27a/b-3p might reduce the protective role of testosterone against cardiovascular disease^14^,^15^. This situation might be of particular relevance in men with low testosterone levels in relation with age or pathologies such as hypogonadism. Indeed, we hypothesized that miR-27a/b-3p may be upregulated in the plasma of men with low concentration of testosterone and TFPI^13^ in comparison with men with normal levels. Results showed no differences in miR-27a/b-3p expression that may be due to a substantial overlap of TFPI expression between the 2 groups (Fig. 7). Data from higher sample size will help to elucidate the role that miR-27a/b-3p may play in the testosterone mediated downregulation of TFPI *in vivo*, and thus whether it could be a contributing factor to the risk of thrombosis associated with low testosterone levels^14^,^15^. In an attempt to further demonstrate the regulation of TFPI by miR-27a/b-3p through testosterone, we compared the levels of miR-27a/b-3p and *TFPI* mRNA in HUVECs in both genders and found no differences (Figure S5). This can be due because at delivery, even if testosterone levels are higher in boys than in girls these levels are 10 times lower than those found in adult individuals^32^. Thus, the regulatory role of testosterone in newborns may be probably negligible.

**Figure 8:**
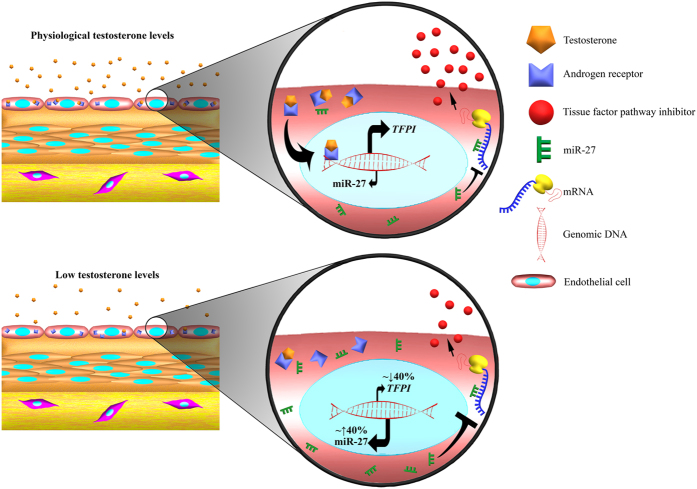
Schematic diagram of the TFPI regulatory mechanism by miRNAs. In the presence of normal levels of testosterone, TFPI expression is regulated in part by the presence of miR-27a/b in the cytosolic compartment. In physiological or pathological conditions where testosterone levels decrease, TFPI expression is downregulated due to a lower transcription of TFPI mRNA but also to a higher repression by miR-27a/b.

In conclusion, our results showed that miRNAs represent a novel mechanism by which TFPIα, a relevant haemostatic molecule, is modulated in ECs. Further *in vivo* studies must be performed to reveal the real transcendence of our results. We suggest that testosterone may be a hinge in miR-27a/b-3p-TFPIα regulation axis (Fig. 8). Given the high variability of miRNA expression levels among healthy population in different tissues, the pathophysiological consequences of this regulation have yet to be established i.e., risk of cardiovascular disease^33^,^34^. In addition, the relevance of our experimental data should be further determined in the context of a potential use of miRNA inhibitors as anti-thrombotic drug.

## Methods

### *In silico* analysis of TFPI 3′UTR

To identify miRNA-binding sites located in human *TFPI* 3′UTR (NM_006287) an *in silico* search using miRNA binding sites prediction programs was performed: miRSVR (http://www.microrna.org), TargetScan (http://www.targetscan.org), and miRWalk (http://www.umm.uni-heidelberg.de/apps/zmf/mirwalk/). MiRWalk covers five different algorithms (DIANA, miRDB, PITA, RNA22, and PICTAR).

### Cell line and tissue samples

Endothelial cell line EA.hy926 was obtained from the American Type Culture Collection (Manassas, VA). ECs were maintained in phenol-red free DMEM supplemented with 2 mM glutamine, and 10% fetal bovine serum (Life Technologies, Madrid, Spain). Human colon cancer cell line HCT-116 deficient for Dicer (HCT-DK) was a kind gift from Dr. Renato Baserga (Thomas Jefferson University, PA). HCT-DK were cultured in McCoy’s 5A (Sigma-Aldrich, Madrid, Spain) supplemented with 2 mM glutamine and 10% fetal bovine serum. Umbilical cord samples were obtained from healthy donors that gave informed consent (La Fe Hospital, Valencia, Spain) (n = 74). Studies with human HUVECs were approved by Local Ethics Committee from Hospital Universitario Morales Meseguer in Murcia and performed according to the declaration of Helsinki. Primary cultures of HUVECs were obtained by standard procedures. Briefly, HUVECs were obtained by collagenase digestion from umbilical cords from healthy donors, and were cultured at 37 °C in humidified air containing 5% CO_2_. The selection of ECs was performed by filtration. HUVECs were grown to confluence in T-75 flasks precoated with endothelial cell attachment factor (Sigma-Aldrich, Madrid, Spain), in medium M199 1X (+) Earle’s, 2mM L-glutamine, 25 mM HEPES, L-aminoacids (Life Technologies, Madrid, Spain), supplemented with 20% fetal bovine serum (Life Technologies, Madrid, Spain), 1% endothelial cell growth factor (ECGF) (Sigma-Aldrich, Madrid, Spain), 1 mM sodium pyruvate, 50 U/mL penicillin, and 50 μg/mL streptomycin sulphate (Life Technologies, Madrid, Spain). Confluent EC monolayers were harvested from the culture flasks with 0.25% trypsin/EDTA solution/0.02% PBS (Biochrom, Cambridge, UK). Cells to be used for total RNA isolation were stored at −80 °C after adding 5 volumes of RNAlater (Life Technologies, Madrid, Spain). Although we had no direct confirmation that the isolated HUVECs were a pure endothelial culture, each culture was examined using a LEITZ DM-IRB inverted fluorescence research microscope (LEICA, Wetzlar, Germany) to ensure that the morphology was consistent with ECs. In all cases cells were cultured under conditions favoring EC growth (medium supplemented with ECGF) and showed the same growth pattern: endothelial-like morphology like as cobblestone-shape area, spindle-shaped cell with cytoplasm distributed homogeneously, without presence of hyperplasia or hill-and-valley morphology, typical for vascular smooth muscle cells culture.

Cells were used within 2–3 passages for all experiments. All the cells were grown at 37 °C under 5% CO_2_ atmosphere.

### Endothelial cells transfections

EA.hy926 and HUVECs were seeded at 50,000/well and transfected with 100 nmol/L miRNA mimics (miR-27a/b-3p, miR-24, miR-19b or scrambled control -SCR-) from Life Technologies (Madrid, Spain) and anti-miRNA inhibitors (anti-miR-27a/b-3p or anti-SCR control) from Exiqon (Vedbaek, Denmark) using siPORT™ *NeoFX™* (Life Technologies, Madrid, Spain) according to manufacturer’s instructions. After 48 h, cells and supernatants were collected for subsequent mRNA and protein analyses. Expression levels of miRNAs after transfection are shown in Supplemental Figure S6.

### Total RNA isolation and qRT-PCR

Total RNA, including miRNAs, was isolated from EA.hy926 and HUVEC with RNAzol Reagent (Molecular Research Center Inc, Cincinnati, OH) according to manufacturer’s instructions. Isolation of miRNA from plasma was performed with NucleoSpin^®^ miRNA plasma kit (Machery-Nagel, Düren, Germany). Reverse transcription (RT) reaction of total RNA (400 ng) was performed using SuperScript™ III First-Strand Synthesis System (Invitrogen, Madrid, Spain). MiRNA cDNA was synthesized from total RNA purified from cells (200 ng) and from plasma (8 μL of each sample) using individual miRNA-specific RT primers and the TaqMan^®^ MicroRNA Reverse Transcription Kit (Life Technologies, Madrid, Spain). Each cDNA was amplified using the TaqMan^®^ MicroRNA assays together with TaqMan^®^ Universal PCR Master Mix, No AmpErase^®^ UNG (Life Technologies, Madrid, Spain). The 2^−ΔCt^ method was followed to calculate the relative abundance of miRNA and mRNA compared with endogenous control expression, U6 for cells miRNA and *ACTB* for mRNA. Plasma miRNA was normalized with geometric mean of miR-103-3p and miR-191 (Ct = Threshold Cycle; ΔCt = Ct sample gene-Ct endogenous control).

### Western blotting and antibodies

Cell supernatants were collected from 24-well plates and BCA assays (ThermoFisher Scientific, Madrid, Spain) were performed to calculate protein concentration of samples. Protein concentration variability between samples was low (4.8–5.5 μg/μL for EAhy.926 and 1.3-1.6 μg/μL for HUVEC). Normalization in the western blot was based on the amount of protein loaded. For this purpose, 100 μg (EAhy.926) and 30 μg (HUVEC) of total protein was separated by SDS-PAGE in reducing conditions by electrophoresis in 8% polyacrylamide gels. Gels were transferred onto PVDF membranes (Amersham Hybond P 0.45, GE Healthcare, Barcelona Spain) and blocked 1 hour in 5% w/v non-fat drymilk. Antibody against human TPFI (#ADG72, Sekisui Diagnostics, Rüsselsheim, Germany) was used 1:5,000 dilution and incubated overnight at 4 °C. The secondary antibody labeled with peroxidase (GE Healthcare, Barcelona, Spain) was used 1:10,000. ECL Prime Detection Kit and ImageQuant LAS 4000 Imager (GE Healthcare, Barcelona, Spain) were used for sample detection. Densitometric analyses were performed with ImageJ software (http://rsb.info.nih.gov/ij/). Linearity between the amount of sample and densitometry was assessed (r^2^ = 0.95). Data were expressed as changes relative to cells transfected with SCR, taken as 100%.

### Plasmid construction and deletion mutagenesis

PCR product (198 bp) containing a fragment of *TFPI* 3′UTR (NM_006287) with miR-27a/b-3p binding site was cloned into pCR 2.1 vector (Life Technologies, Madrid, Spain) using the primers: (5′GAGCTCCGTTATTTTTACCGTGTTTTG and 5′ACGCGTCGTTTGAGTGGTTTTCAG). SacI and MluI were used to digest positive clones (New England Biolabs, Ipswich, MA). The insert was subcloned into luciferase reporter plasmid pMIR-REPORT (Life Technologies, Madrid, Spain) previously digested with these enzymes. A deletion mutant of pMIR-REPORT-TFPI without miR-27a/b-3p seed region-binding site was generated using the primers S: 5′GAGCTCCGTTATTTTTACCGTGTTTTG and AS: 5′ACGCGTCGTTTGAGTGGTTTTCAG with the QuikChange site-directed mutagenesis kit (Agilent Technologies, Santa Clara, CA). Insertion of TFPI 3′ UTR fragment and seed sequence deletion was confirmed by sequencing. PCR amplification was performed on plasmid DNA (Platinum Taq High Fidelity, Life Technologies, Madrid, Spain) and cleaned-up using ExoSAP-IT (Affymetrix, Santa Clara, CA). Sequences were performed using the BigDye Terminator Reaction Chemistry v3.1 on Applied Biosystems 3130 XL DNA analyzers (Life Technologies, Madrid, Spain). Alignments and sequence analysis were performed using Seqman Pro program (Lasergene version 7.1, DNASTAR, Madison, WI).

### Luciferase assay

HCT-DK cells were co-transfected with miR-27a/b-3p (both pMIR-REPORT plasmids −500 ng/well-wild type or mutated for the miR-27a/b-3p seed site) or SCR control, and 50 ng/well of renilla luciferase control plasmid (pRL-TK; Promega, Madison, WI) using Lipofectamine LTX (Life Technologies) according to the manufacturer’s instructions. After transfection (48 h), firefly and renilla luciferase activities were measured using the Dual-Glo Luciferase Reporter Assay (Promega, Madison, WI) in a Synergy 2 luminometer (Biotek, Winooski, VT).

### TFPIα functional assay

The inhibitory activity of TFPIα was measured by functional chromogenic assay on supernatants from transfected cells (EAhy.926 and HUVECs), where TF-FVIIa dependent FXa generation was quantified using Actichrome^®^ TFPI activity assay kit (Sekisui Diagnostics, Rüsselsheim, Germany) following manufacturer’s protocol. Briefly, 20 μL of culture supernatant (adjusted by BCA method to optimal concentration depending on cell line) were added into the wells of a micro-test plate. Then, 20 μL of FVIIa/TF complex were added and incubated at 37 °C for 30 min. After that, 20 μL of human FX were added into each well and incubated for 15 min. After adding 20 μL of EDTA, the reaction was initiated by adding 20 μL of spectrozyme FXa substrate. Absorbance was read at 405 nm in a spectrophotometer for 10 min and Vmax was calculated.

### Endothelial cells treatment

EA.hy926 and HUVEC cells pre-incubated for 48 h with 10% steroid-free FBS (charcoal/dextran-stripped serum from GE Hyclone, Logan, UT) were treated for 24 h with 15 nM or 30 nM (physiologic concentration) dihydrotestosterone (DHT) (Sigma-Aldrich, Madrid, Spain) or 0.1% ethanol (vehicle control).

### Statistical analysis

Statistical analysis was performed with SPSS 21.0 (SPSS, Inc., Chicago, IL, USA) and GraphPad Prism 3.0 (GraphPad Software, Inc., La Jolla, CA, USA). For two groups comparisons, non-parametric test (U Mann–Whitney) was used. For multiple comparisons, one-way ANOVA on ranks with a post-hoc test (Bonferroni correction) were used. Association between continuous variables was analyzed by the Spearman’s rank correlation coefficient. Normal distribution of each variable was analyzed using one sample Kolmogorov-Smirnov test with Lilliefors significance correction. Results were expressed as mean ± SD. All tests were two-sided, and statistical significance was defined as a *p*-value < 0.05 (or smaller for corrected *p*-values in multiple comparisons).

## Additional Information

**How to cite this article**: Arroyo, A.B. *et al*. Regulation of TFPIα expression by miR-27a/b-3p in human endothelial cells under normal conditions and in response to androgens. *Sci. Rep.* **7**, 43500; doi: 10.1038/srep43500 (2017).

**Publisher's note:** Springer Nature remains neutral with regard to jurisdictional claims in published maps and institutional affiliations.

## Supplementary Material

Supplementary Data

## Figures and Tables

**Table 1:** *In silico* search of potential miRNAs able to bind to 3′ UTR of *TFPI*.

		TargetScan score	miRanda miRSVR score	miRWalk				
3′ UTR of TFPI/miRNA alignment				DIANAmt	miRDB	PITA	RNA22	PICTAR5
Position 2546-2553 of TFPI 3′ UTR hsa-miR-27a-3p	5′ …AAUUAAGUGCAUCUAACUGUGAA… ||||||| 3′ CGCCUUGAAUCGGUGACACUU	−0.36	−0.99	+	+	−	−	+
Position 2546-2553 of TFPI 3′ UTR hsa-miR-27b-3p	5′ …AAUUAAGUGCAUCUAACUGUGAA… ||||||| 3′ CGUCUUGAAUCGGUGACACUU	−0.36	−0.99	+	+	−	−	+
Position 865-872 of TFPI 3′ UTR hsa-miR-19	5′ …CAGUUAUCAUUAGGAUUUGCACA… ||||||| 3′ AGUCAAAACGUACCUAAACGUGU	−0.15	−0.63	+	+	+	−	−
Position 1040-1047 of TFPI 3′ UTR hsa-miR-24	5′ …AUCUUUCAUUUAUUGCUGAGCCA… ||||||| 3′ GACAAGGACGACUUGACUCGGU	−0.15	−0.53	+	+	−	−	+

## References

[b1] Winckers K., ten C. H. & Hackeng T. M. The role of tissue factor pathway inhibitor in atherosclerosis and arterial thrombosis. Blood Rev. 27, 119–132 (2013). 23631910 10.1016/j.blre.2013.03.001

[b2] Almasy L. . A locus on chromosome 2 influences levels of tissue factor pathway inhibitor: results from the GAIT study. Arterioscler. Thromb. Vasc. Biol. 25, 1489–1492 (2005). 15845911 10.1161/01.ATV.0000166602.04711.2e

[b3] Warren D. M. . Heritability of hemostasis phenotypes and their correlation with type 2 diabetes status in Mexican Americans. Hum. Biol. 77, 1–15 (2005). 16114812 10.1353/hub.2005.0034

[b4] Bladbjerg E. M. . Genetic influence on thrombotic risk markers in the elderly–a Danish twin study. J. Thromb. Haemost. 4, 599–607 (2006). 16371117 10.1111/j.1538-7836.2005.01778.x

[b5] Dennis J., Kassam I., Morange P. E., Tregouet D. A. & Gagnon F. Genetic determinants of tissue factor pathway inhibitor plasma levels. Thromb. Haemost. 114, 245–257 (2015). 25879386 10.1160/TH14-12-1043

[b6] Teruel-Montoya R., Rosendaal F. R. & Martinez C. MicroRNAs in hemostasis. J. Thromb. Haemost. 13, 170–181 (2015). 25400249 10.1111/jth.12788

[b7] Teruel R. . Identification of miRNAs as potential modulators of tissue factor expression in patients with systemic lupus erythematosus and antiphospholipid syndrome. J. Thromb. Haemost. 9, 1985–1992 (2011). 21794077 10.1111/j.1538-7836.2011.04451.x

[b8] Bartel D. P. MicroRNAs: target recognition and regulatory functions. Cell 136, 215–233 (2009). 19167326 10.1016/j.cell.2009.01.002 PMC3794896

[b9] Van R. K., Pollet J. & Calin G. A. miRNAs and long noncoding RNAs as biomarkers in human diseases. Expert. Rev. Mol. Diagn. 13, 183–204 (2013). 23477558 10.1586/erm.12.134

[b10] Quiat D. & Olson E. N. MicroRNAs in cardiovascular disease: from pathogenesis to prevention and treatment. J. Clin. Invest. 123, 11–18 (2013). 23281405 10.1172/JCI62876 PMC3533276

[b11] Jin H. . Physiological testosterone stimulates tissue plasminogen activator and tissue factor pathway inhibitor and inhibits plasminogen activator inhibitor type 1 release in endothelial cells. Biochem. Cell Biol. 85, 246–251 (2007). 17534406 10.1139/O07-011

[b12] Lupu C., Zhu H., Popescu N. I., Wren J. D. & Lupu F. Novel protein ADTRP regulates TFPI expression and function in human endothelial cells in normal conditions and in response to androgen. Blood 118, 4463–4471 (2011). 21868574 10.1182/blood-2011-05-355370 PMC3204913

[b13] Agledahl I., Brodin E., Svartberg J. & Hansen J. B. Plasma free tissue factor pathway inhibitor (TFPI) levels and TF-induced thrombin generation *ex vivo* in men with low testosterone levels. Thromb. Haemost. 101, 471–477 (2009). 19277407

[b14] Khaw K. T. . Endogenous testosterone and mortality due to all causes, cardiovascular disease, and cancer in men: European prospective investigation into cancer in Norfolk (EPIC-Norfolk) Prospective Population Study. Circulation 116, 2694–2701 (2007). 18040028 10.1161/CIRCULATIONAHA.107.719005

[b15] Haring R. . Low serum testosterone levels are associated with increased risk of mortality in a population-based cohort of men aged 20-79. Eur. Heart J. 31, 1494–1501 (2010). 20164245 10.1093/eurheartj/ehq009

[b16] Waltering K. K. . Androgen regulation of micro-RNAs in prostate cancer. Prostate 71, 604–614 (2011). 20945501 10.1002/pros.21276

[b17] Wang W. L., Chatterjee N., Chittur S. V., Welsh J. & Tenniswood M. P. Effects of 1alpha, 25 dihydroxyvitamin D3 and testosterone on miRNA and mRNA expression in LNCaP cells. Mol. Cancer 10, 58 (2011). 21592394 10.1186/1476-4598-10-58 PMC3112430

[b18] Narayanan R. . MicroRNAs are mediators of androgen action in prostate and muscle. PLoS One 5, e13637 (2010). 21048966 10.1371/journal.pone.0013637 PMC2965097

[b19] Nielsen S. . Muscle specific miRNAs are induced by testosterone and independently upregulated by age. Front. Physiol. 4, 394 (2013). 24478708 10.3389/fphys.2013.00394 PMC3899547

[b20] Li Y. . Comprehensive analysis of the functional microRNA-mRNA regulatory network identifies miRNA signatures associated with glioma malignant progression. Nucleic Acids Res. 41, e203 (2013). 24194606 10.1093/nar/gkt1054 PMC3905890

[b21] Hackeng T. M., Sere K. M., Tans G. & Rosing J. Protein S stimulates inhibition of the tissue factor pathway by tissue factor pathway inhibitor. Proc. Natl. Acad. Sci. USA 103, 3106–3111 (2006). 16488980 10.1073/pnas.0504240103 PMC1413864

[b22] Tay J. W., Romeo G., Hughes Q. W. & Baker R. I. Micro-Ribonucleic Acid 494 regulation of protein S expression. J. Thromb. Haemost. 11, 1547–1555 (2013). 23789915 10.1111/jth.12331

[b23] Vickers K. C. . MicroRNA-27b is a regulatory hub in lipid metabolism and is altered in dyslipidemia. Hepatology 57, 533–542 (2013). 22777896 10.1002/hep.25846 PMC3470747

[b24] Kuehbacher A., Urbich C., Zeiher A. M. & Dimmeler S. Role of Dicer and Drosha for endothelial microRNA expression and angiogenesis. Circ. Res. 101, 59–68 (2007). 17540974 10.1161/CIRCRESAHA.107.153916

[b25] Mendell J. T. & Olson E. N. MicroRNAs in stress signaling and human disease. Cell 148, 1172–1187 (2012). 22424228 10.1016/j.cell.2012.02.005 PMC3308137

[b26] Lin Q., Gao Z., Alarcon R. M., Ye J. & Yun Z. A role of miR-27 in the regulation of adipogenesis. FEBS J. 276, 2348–2358 (2009). 19348006 10.1111/j.1742-4658.2009.06967.x PMC5330386

[b27] Buck A. H. . Post-transcriptional regulation of miR-27 in murine cytomegalovirus infection. RNA. 16, 307–315 (2010). 20047990 10.1261/rna.1819210 PMC2811660

[b28] Xie N. . miR-27a regulates inflammatory response of macrophages by targeting IL-10. J. Immunol. 193, 327–334 (2014). 24835395 10.4049/jimmunol.1400203 PMC4065847

[b29] Maroney S. A. & Mast A. E. Tissue factor pathway inhibitor and bacterial infection. J. Thromb. Haemost. 9, 119–121 (2011). 21210950 10.1111/j.1538-7836.2010.04111.x PMC3034136

[b30] Fletcher C. E. . Androgen-regulated processing of the oncomir miR-27a, which targets Prohibitin in prostate cancer. Hum. Mol. Genet. 21, 3112–3127 (2012). 22505583 10.1093/hmg/dds139

[b31] Pomerantz M. M. . The androgen receptor cistrome is extensively reprogrammed in human prostate tumorigenesis. Nat. Genet. 47, 1346–1351 (2015). 26457646 10.1038/ng.3419 PMC4707683

[b32] Tomlinson C. . Testosterone measurements in early infancy. Arch. Dis. Child. Fetal Neonatal Ed. 89, F558–F559 (2004). 15499155 10.1136/adc.2003.034017 PMC1721770

[b33] Teruel-Montoya R. . MicroRNA expression differences in human hematopoietic cell lineages enable regulated transgene expression. PLoS One 9, e102259 (2014). 25029370 10.1371/journal.pone.0102259 PMC4100820

[b34] Salloum-Asfar S. . Regulation of Coagulation Factor XI Expression by MicroRNAs in the Human Liver. PLoS One 9, e111713 (2014). 25379760 10.1371/journal.pone.0111713 PMC4224396

