# Supplementary material for: Regulation of TFPIα expression by miR-27a/b-3p in human endothelial cells under normal conditions and in response to androgens
Source: Sci Rep. 2017 Feb 27;7:43500. doi: 10.1038/srep43500 (PMC5327489; doi:10.1038/srep43500)
Supplement: Supplementary Data [file srep43500-s1.doc]

**Supplemental Figures**

**Regulation of TFPIα expression by miR-27a/b-3p in human endothelial cells under normal conditions and in response to androgens.**

Ana Belén Arroyo, Salam Salloum-Asfar, Carlos Pérez-Sánchez, Raúl Teruel-Montoya, Silvia Navarro, Nuria García-Barberá, Ginés Luengo-Gil, Vanessa Roldán, John-Bjarne Hansen, Chary López-Pedrera, Vicente Vicente, Rocío González-Conejero, Constantino Martínez


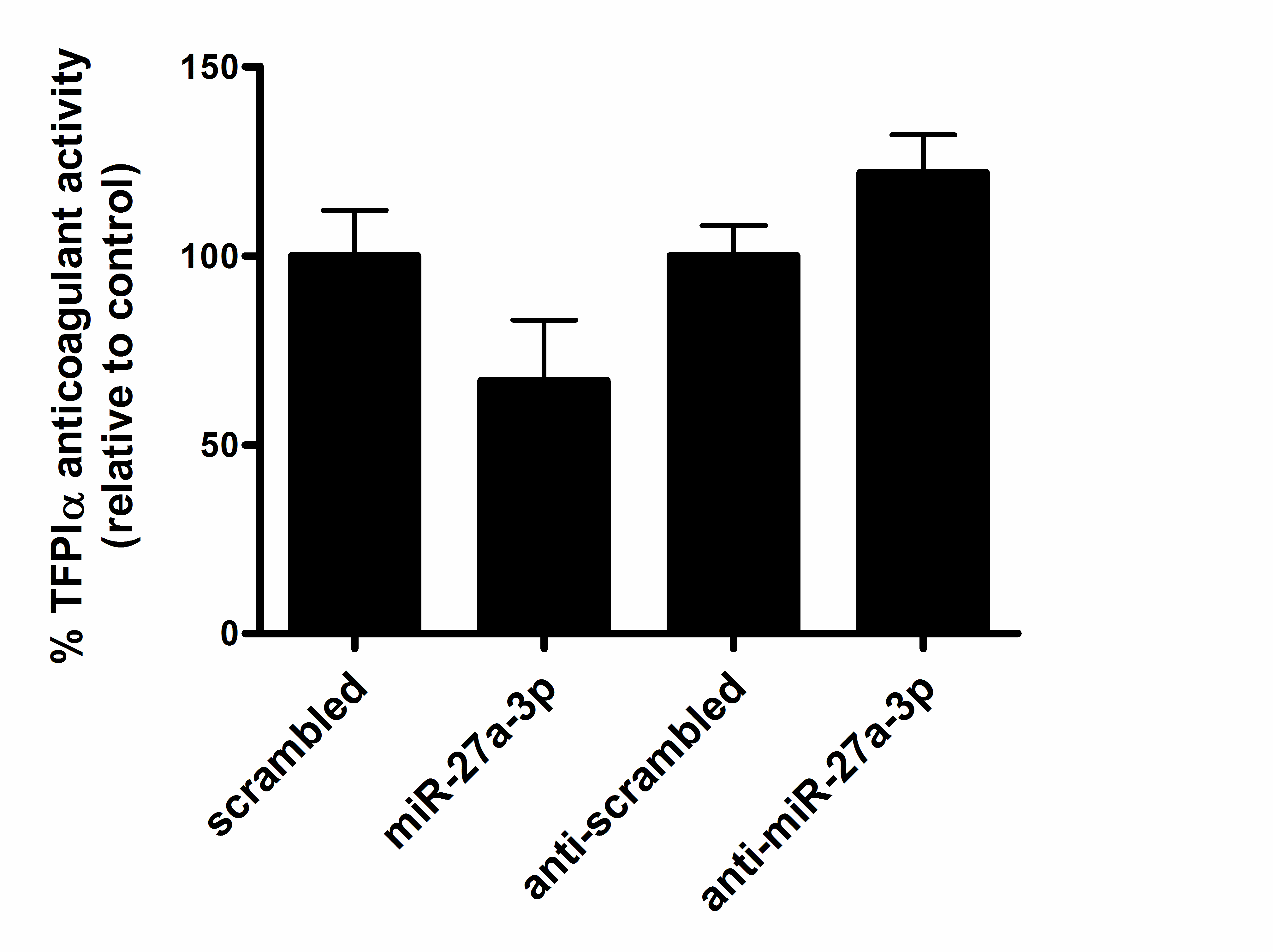


**Figure S1. miR-27a regulates TFPI anticoagulant activity.** TFPIα anticoagulant activity was measured in the culture medium from EA.hy926 cells transfected with precursors or inhibitors of miR-27a-3p. Results are represented as mean ± SD from two experiments performed in triplicate.

**A)**


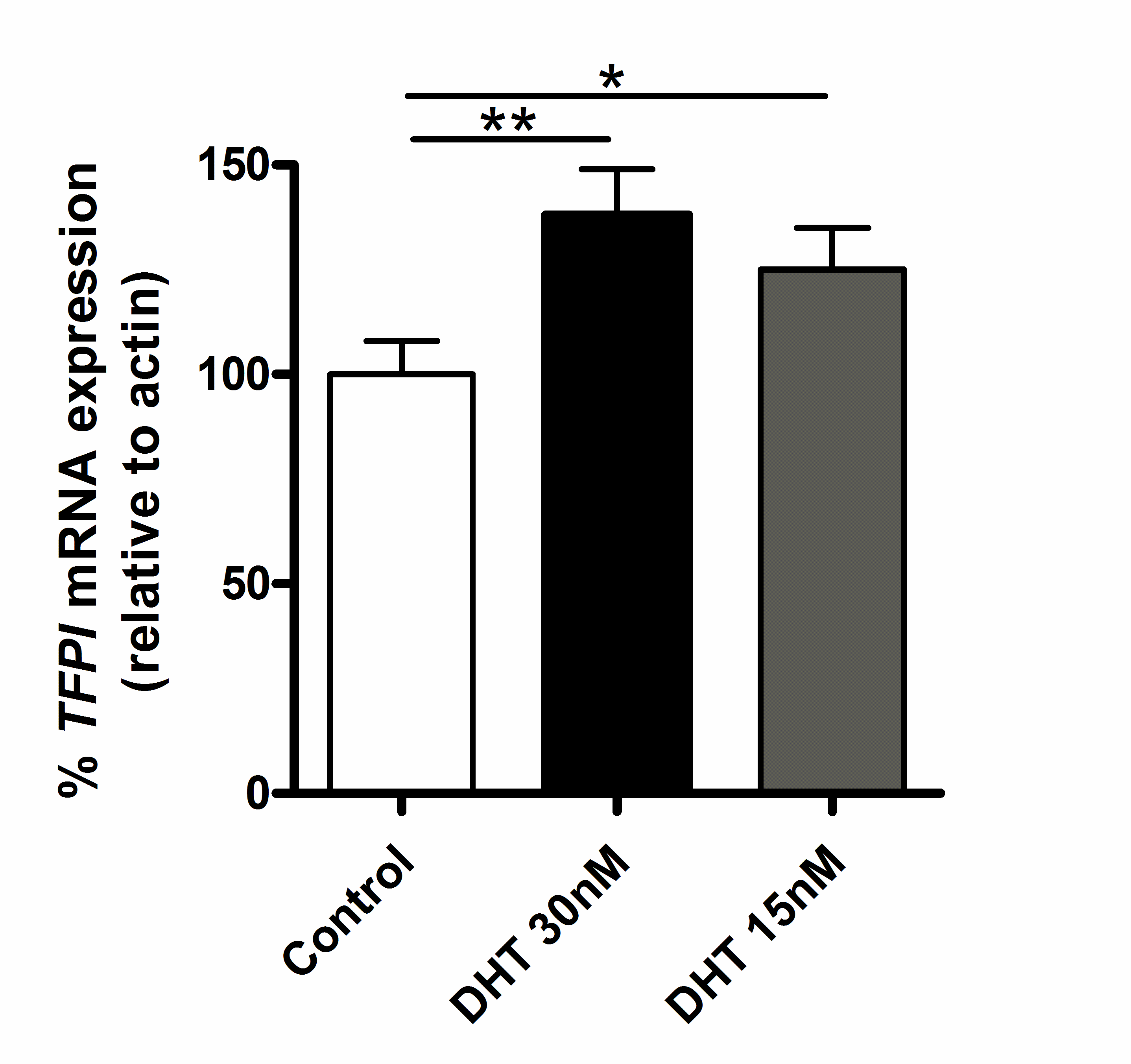


**B) C)**

**
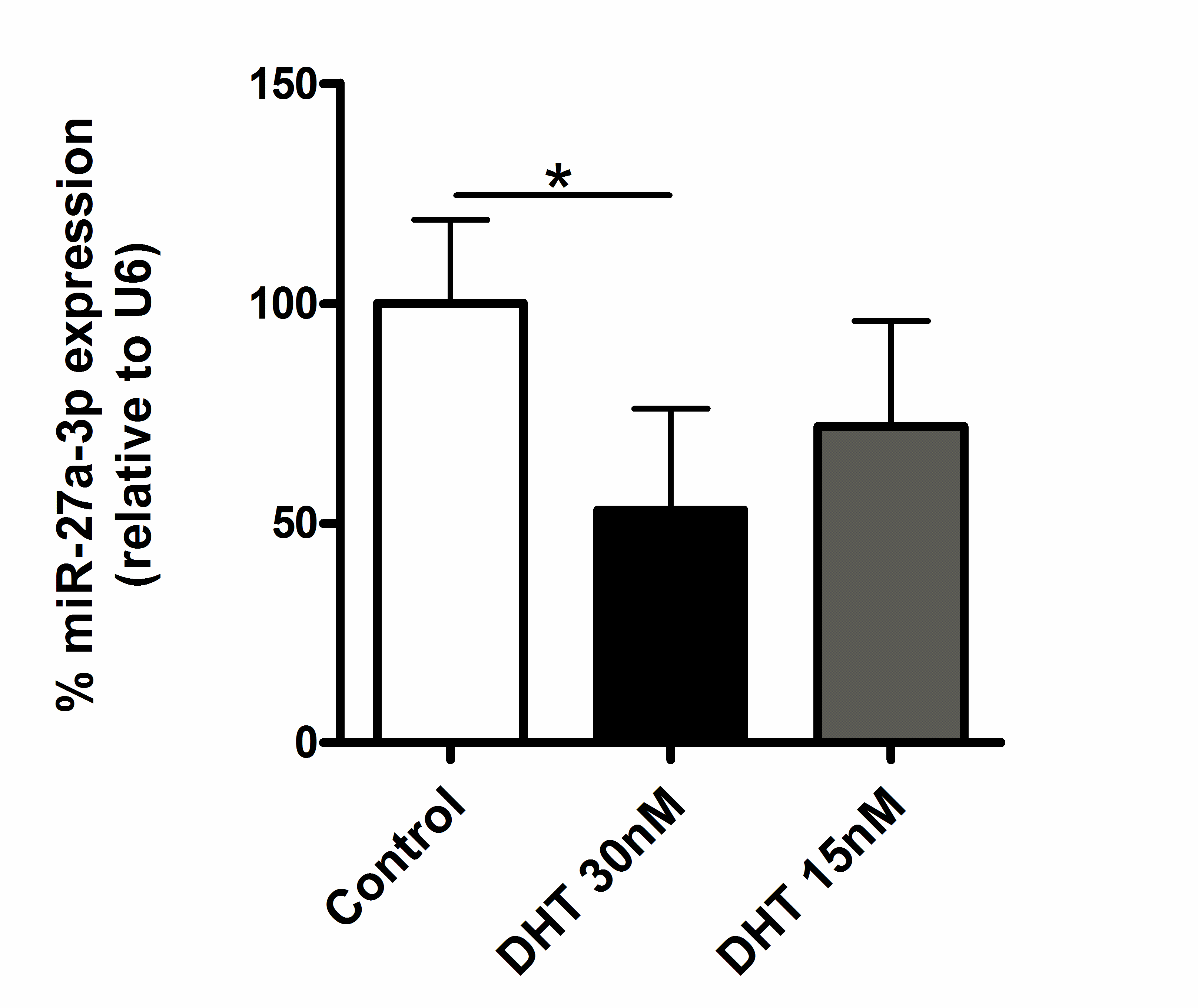

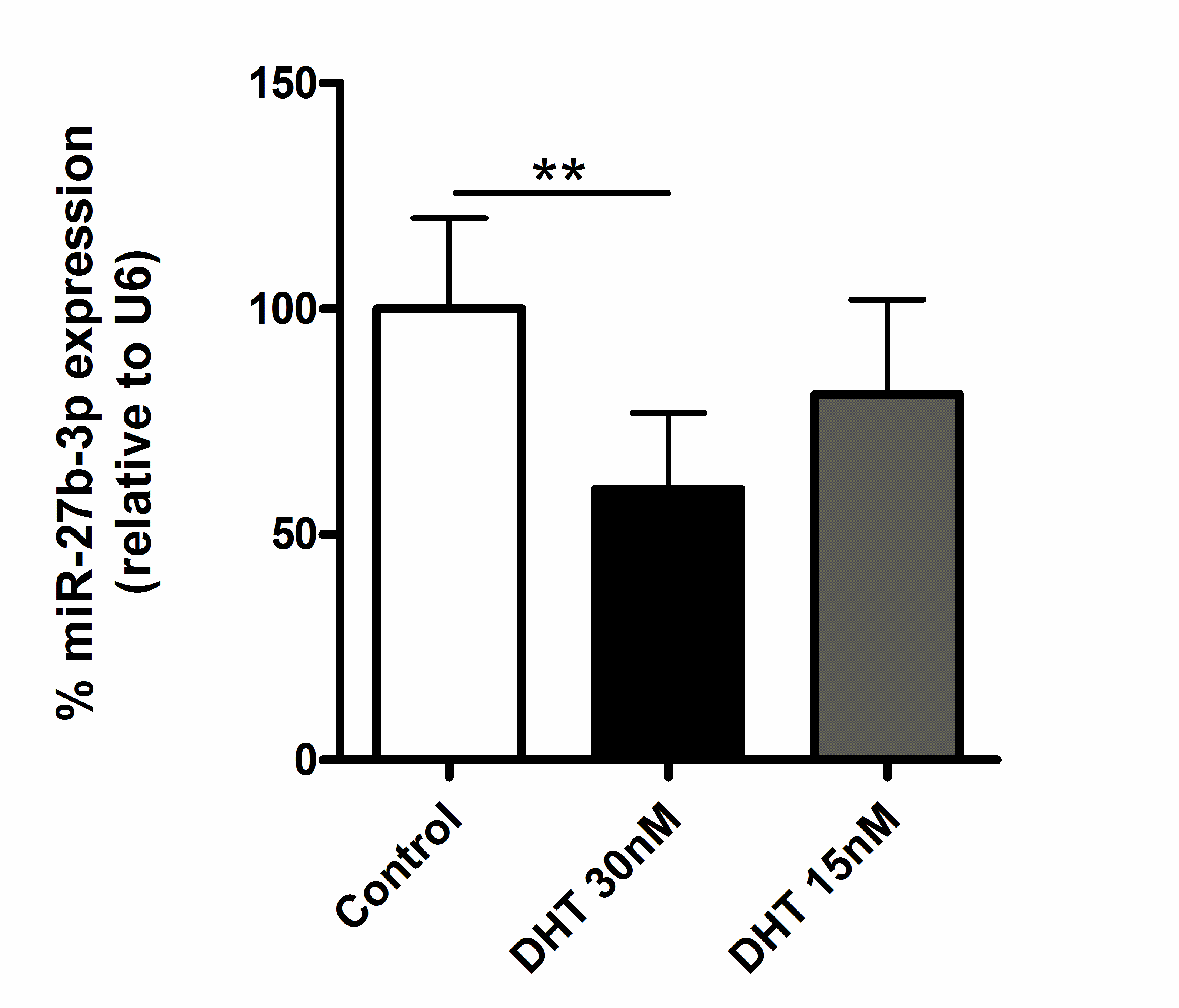
**

**Figure S2. DHT regulates miR-27a/b-3p and *TFPI* expression in HUVECs.**

HUVECs were activated with physiological (30 nM) or low (15 nM) doses of DHT and *TFPI* mRNA (A) and miR-27a/b-3p levels (B and C) were measured by qRT-PCR. The 2^-ΔCt^ method was followed to calculate the relative abundance of miRNA or mRNA compared with endogenous control expression of U6 or *ACTB* (Ct=Threshold Cycle; ΔCt = Ct sample gene - Ct endogenous control). All results are represented as mean ± SD from at least three experiments performed in triplicate (*p<0.05; **p<0.01).

**
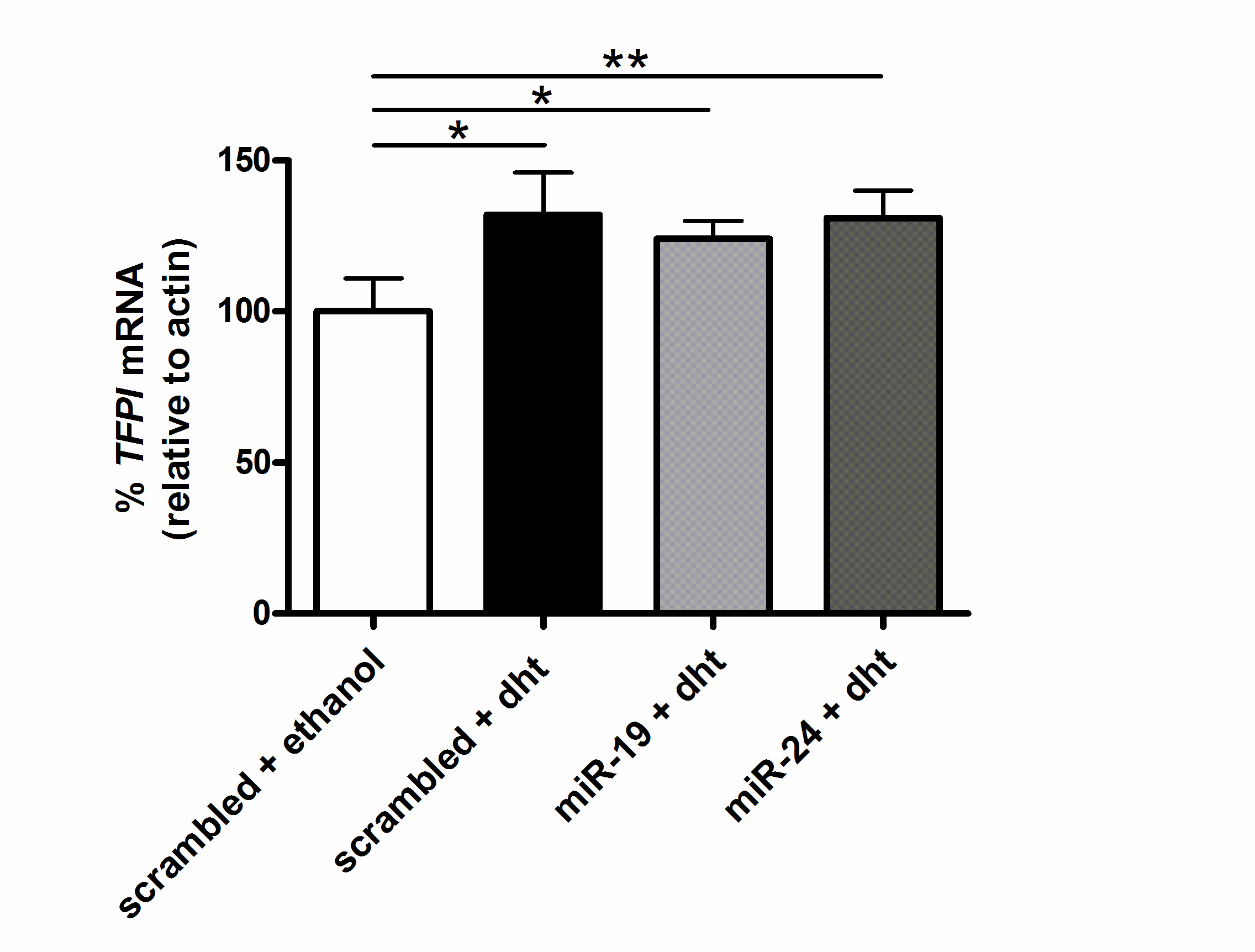
**

**Figure S3. MiR-19b and miR-24 do not affect *TFPI* induced expression by testosterone**. EA.hy926 cells transfected with miR-19b or miR-24 precursors were activated with 30 nM DHT. After 48h levels of *TFPI* mRNA were measured by qRT-PCR. The 2^-ΔCt^ method was followed to calculate the relative abundance *TFPI* mRNA compared with endogenous control expression of *ACTB* (Ct=Threshold Cycle; ΔCt = Ct sample gene - Ct endogenous control). All results are represented as mean ± SD from at least three experiments performed in triplicate (*p<0.05; **p<0.01).

**
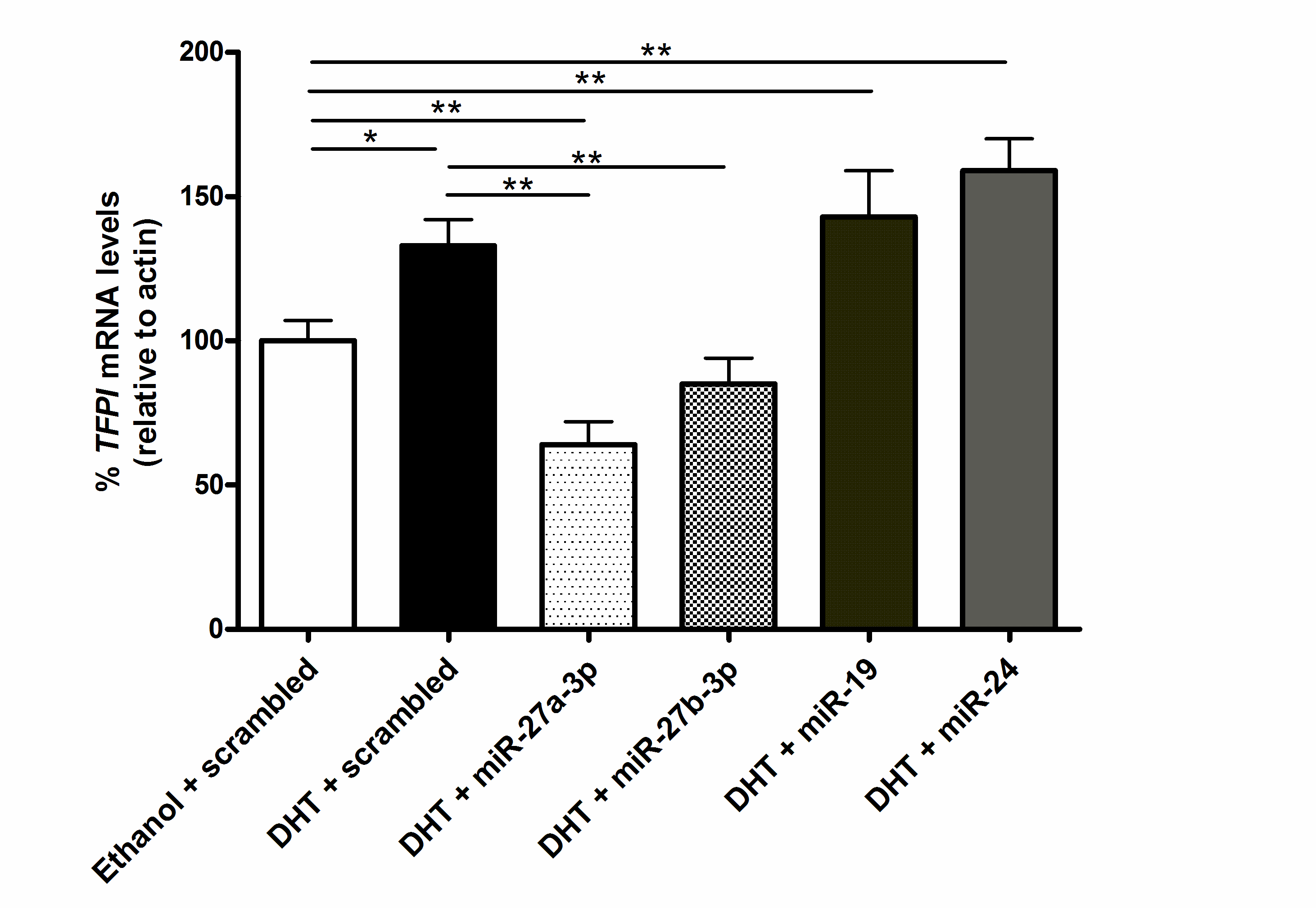
**

**Figure S4. Only miR-27a/b-3p affect *TFPI* mRNA induced expression by testosterone**. HUVEC cells transfected with miR-27a/b-3p, miR-19b or miR-24 precursors were activated with 30 nM DHT. After 48h levels of *TFPI* mRNA were measured by qRT-PCR. The 2^-ΔCt^ method was followed to calculate the relative abundance *TFPI* mRNA compared with endogenous control expression of *ACTB* (Ct=Threshold Cycle; ΔCt = Ct sample gene - Ct endogenous control). All results are represented as mean ± SD from at least three experiments performed in triplicate (*p<0.05;**p<0.01).


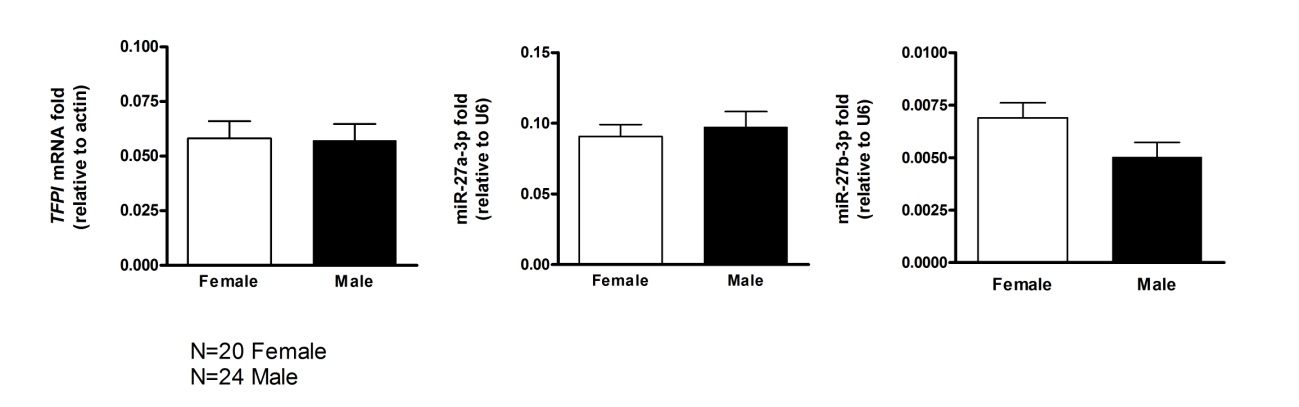
**Figure S5. Levels of miR-27a/b-3p and TFPI in HUVECs by gender.** Levels of miR-27a-3p, miR-27b-3p, and *TFPI* mRNA were measured by qRT-PCR. The 2^-ΔCt^ method was followed to calculate the relative abundance of miRNA compared with endogenous control expression of U6 and *ACTB* (Ct=Threshold Cycle; ΔCt = Ct sample gene - Ct endogenous control).


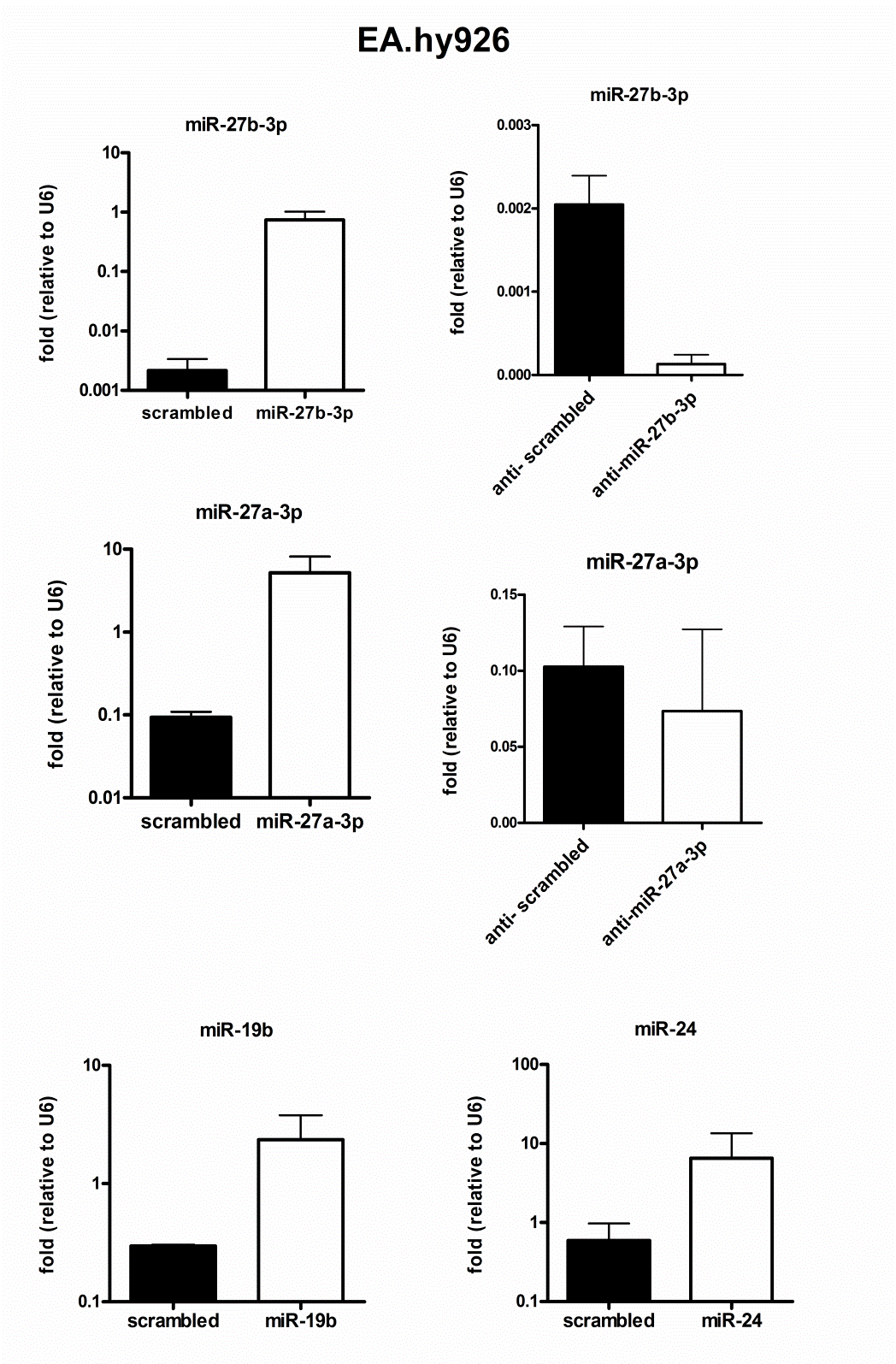


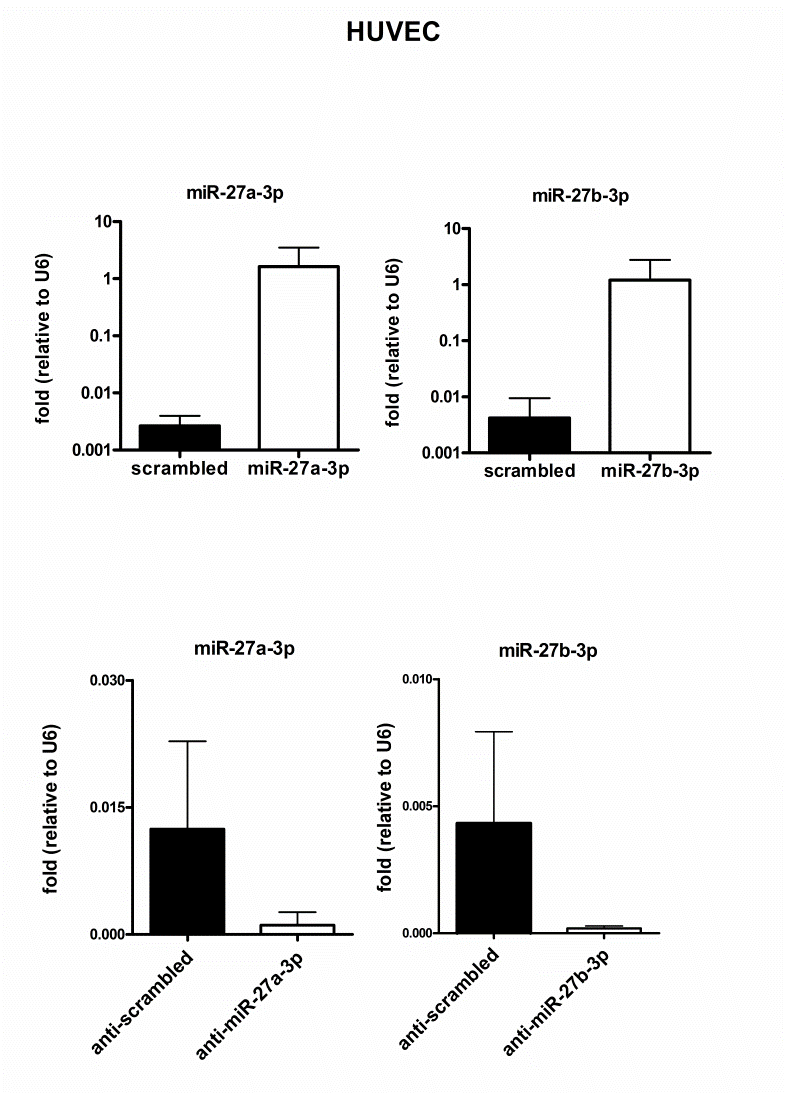


**Figure S6. Levels of miRNA after transfection with mimics or inhibitors.** Levels of miR-27a-3p, miR-27b-3p, miR-19b, and miR-24 were measured after transfection of EAhy.926 cells. The 2^-ΔCt^ method was followed to calculate the relative abundance of miRNA compared with endogenous control expression of U6 (Ct=Threshold Cycle; ΔCt = Ct sample gene - Ct endogenous control).
